# The conservation pattern of short linear motifs is highly correlated with the function of interacting protein domains

**DOI:** 10.1186/1471-2164-9-452

**Published:** 2008-10-01

**Authors:** Siyuan Ren, Guang Yang, Youyu He, Yiguo Wang, Yixue Li, Zhengjun Chen

**Affiliations:** 1 State Key Laboratory of Molecular Biology, Institute of Biochemistry and Cell Biology, Chinese Academy of Sciences, Shanghai, PR China; 2 Bioinformatics Center, Shanghai Institutes for Biological Sciences, Chinese Academy of Sciences, Shanghai, PR China; 3 Center for Information Science and Technology, Temple University, Philadelphia, PA 19122, USA

## Abstract

**Background:**

Many well-represented domains recognize primary sequences usually less than 10 amino acids in length, called Short Linear Motifs (SLiMs). Accurate prediction of SLiMs has been difficult because they are short (often < 10 amino acids) and highly degenerate. In this study, we combined scoring matrixes derived from peptide library and conservation analysis to identify protein classes enriched of functional SLiMs recognized by SH2, SH3, PDZ and S/T kinase domains.

**Results:**

Our combined approach revealed that SLiMs are highly conserved in proteins from functional classes that are known to interact with a specific domain, but that they are not conserved in most other protein groups. We found that SLiMs recognized by SH2 domains were highly conserved in receptor kinases/phosphatases, adaptor molecules, and tyrosine kinases/phosphatases, that SLiMs recognized by SH3 domains were highly conserved in cytoskeletal and cytoskeletal-associated proteins, that SLiMs recognized by PDZ domains were highly conserved in membrane proteins such as channels and receptors, and that SLiMs recognized by S/T kinase domains were highly conserved in adaptor molecules, S/T kinases/phosphatases, and proteins involved in transcription or cell cycle control. We studied Tyr-SLiMs recognized by SH2 domains in more detail, and found that SH2-recognized Tyr-SLiMs on the cytoplasmic side of membrane proteins are more highly conserved than those on the extra-cellular side. Also, we found that SH2-recognized Tyr-SLiMs that are associated with SH3 motifs and a tyrosine kinase phosphorylation motif are more highly conserved.

**Conclusion:**

The interactome of protein domains is reflected by the evolutionary conservation of SLiMs recognized by these domains. Combining scoring matrixes derived from peptide libraries and conservation analysis, we would be able to find those protein groups that are more likely to interact with specific domains.

## Background

Selective protein-protein interactions are important for cellular functions and are often mediated by protein domains that recognize specific primary sequences within target proteins called Short Linear Motifs (SLiMs). Accurate prediction of SLiMs has been difficult because they are short (often < 10 amino acids) and highly degenerate. A major advance in SLiM identification came with a peptide library-based technique that can map the sequence motif recognized by an SH2 domain without prior knowledge of *in vivo *interaction sites [1]. Similar peptide library experiments have been performed to map the motifs recognized by other domains. Motifs discovered through polypeptide library screening have shown high levels of agreement with reported domain interaction sites [1,2]. This became the basis for Scansite [3,4], a bioinformatics program developed to predict SLiMs in query proteins that are recognized by specific protein domains. Other bioinformatic approaches, like those available in Minimotif-Miner [5], QuasiMotifFinder [6], MCS [7] and a tree-based scoring [8] applied evolutionary conservation as well as other sequence filters to assess the functional relevance of a hit.

Both peptide library screening and evolutionary conservation proved to be useful in prediction motifs, we hypothesized that combining chemical enrichment scoring matrixes derived from peptide libraries and conservation analysis would discriminate between classes of proteins that have functional SLiMs and those that do not. To address this issue, we conducted a global statistical analysis on the conservation of SLiMs recognized by SH2, SH3, PDZ and S/T kinase domains (Invariant features in SLiMs recognized by each domain were shown in Table 1) in different functional classes of proteins. For each domain we studied, our analysis revealed that domain-recognized SLiMs are highly conserved in specific functional classes of proteins that are known to frequently interact with that domain, but they are not conserved in most other protein groups. For example, we found that SLiMs that interact with SH2 domains are conserved in receptor kinases/phosphatases, adaptor molecules, and tyrosine kinases/phosphatases. Our analysis also confirmed that most SH2-mediated signaling occurs in the cytoplasm, and suggests that SLiMs that are recognized by tyrosine kinases and are in proteins that contain multiple SH3 binding motifs are more likely to interact with SH2 domains.

**Table 1:** Invariant features in SLiMs recognized by SH2, SH3, PDZ and S/T Kinase domains

**Domain**	**SLiM**	**length**
SH2	YXXX	4
SH3 Type 1	XXXPXXP	7
SH3 Type 2	XPXXPXX	7
PDZ	XXXXX-COOH	5
S/T Kinase	XXXXS/TXXXX	9

## Results

### Relative Conservation (C_R_) of SLiMs

**Figure 1:**
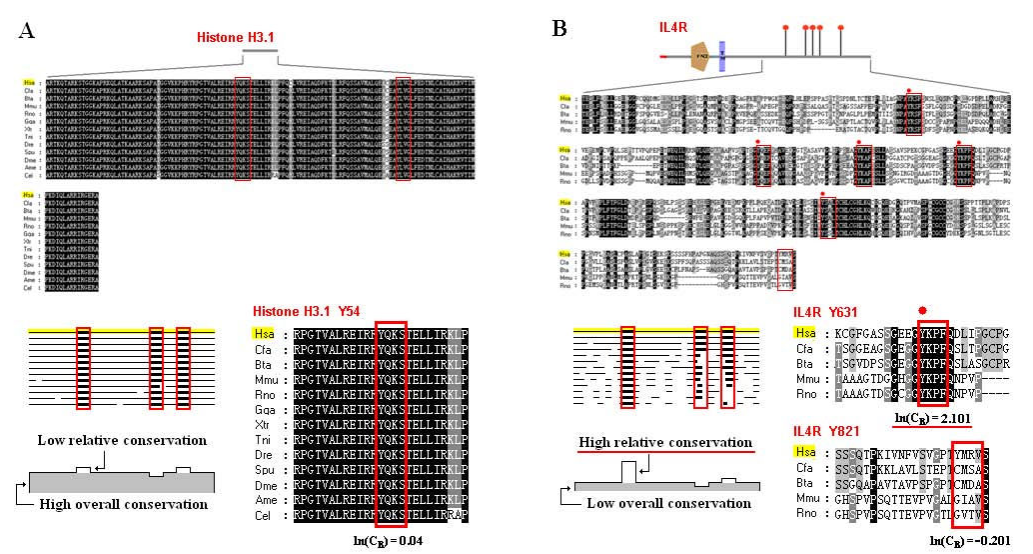
**Relative conservation of SLiMs**. (A) Low relative conservation of a conserved SLiM in an overall highly conserved protein. Sequence alignment of Histone H3.1 with potential SH2 binding Tyr-SLiMs in red boxes (upper panel). Schematic illustration and alignment around Y54 of Histone H3.1 are shown below. (B) High relative conservation of a conserved SLiM in an overall less-conserved protein. C-terminal sequence alignment of IL4R with potential SH2 binding Tyr-SLiMs shown in red boxes (upper panel). Schematic illustration of relative conservation and alignment around Y631 and Y821 of IL4R are shown below.

Relative conservation of SLiMs was measured to assess their functional importance. The central hypothesis was that SLiMs should be subject to two kinds of evolutionary selection. The first is background selection, which is imposed upon the entire length of the protein sequence, and is due to factors such as the overall stability, structure, and function of the protein. The second is SLiM-specific selection superimposed on the background, due to the special function mediated by the SLiM. Therefore, a well-conserved SLiM in an overall highly conserved protein does not guarantee independent importance. For example, although the two putative SH2 binding Tyr-SLiMs in Histone H3.1 were conserved among sequences from all selected species (Figure 1A upper panel), their relative conservation was low because of the highly conserved background (see Figure 1A lower panel for a schematic illustration of the background and SLiM specific relative conservation and alignment of Histone H3.1 Y54). It is possible Tyr-SLiMs in Histone H3.1 are conserved because they have an integral function in protein structure or stability. Conversely, a SLiM with high relative conservation is an indication that the motif may play a unique physiological role. The five Tyr-SLiMs in the C-terminus of IL4R are well conserved, while the full-length protein is not (Figure 1B upper panel); thus, these SLIMs have a high level of relative conservation (see Figure 1B lower panel for a schematic illustration of the relative conservation and alignments of IL4R Y631 and Y821). In fact, four of these five conserved tyrosine motifs are reported to bind to SH2 domains [9]. The relative conservation method allows us to discriminate between SLiMs that have been conserved due to structural constraints of the protein from those that have been conserved to serve as functional motifs. We do not argue against the importance of conserved motifs in conserved proteins; however, we consider them less likely to function independently.

### Analysis of SH2 Domain-Mediated Signaling in 11 Highly Studied Receptor Tyrosine Kinases (RTKs)

**Figure 2:**
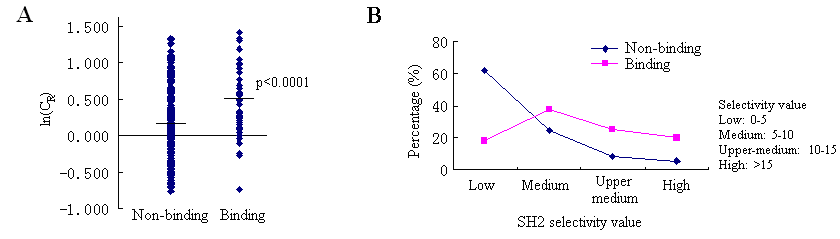
**Comparison of conservation scores and SH2 selectivity values between SH2 binding and non-binding Tyr-SLiMs in 11 highly studied RTKs**. (A) SH2 binding Tyr-SLiMs are significantly more conserved than those that do not bind to SH2 domains (p < 0.0001, Mann-Whitney test). (B) Percentage of SLiMs that have different SH2 selectivity values in binding and non-binding groups.

To test the functional relevance of our SLiM conservation calculation, we analyzed reported SH2 binding sites in 11 highly-studied RTKs (with greater than 30 interaction partners, according to Hprd), including EGFR, IR, KIT, PDGFRB, IGF-IR, VEGFR2, ERBB2, FGFR1, HGFR, RET and TKR-A. We manually extracted interactions from the literature between one of these RTKs and one of the 21 SH2 domains we are studying here, which yielded a total of 76 interactions involving 56 unique Tyr-SLiMs (refer to Table S1 for detail). Using our SLiM conservation calculation, we found that reported SH2 binding sites have significantly higher (p < 0.0001, Mann-Whitney test) ln(C_R_) scores (which measures the relative conservation of a motif) than those sites that do not bind to SH2 domains (Figure 2A), indicating the relative conservation score is an effective distinguishing factor of functional binding SLiMs.

In order to evaluate the specificity of motif prediction, we compared the SH2 selectivity values (which is calculated using enrichment values from peptide library screening) of SLiMs in proteins from reported binding groups to the SH2 selectivity values of SLiMs in proteins from groups that are not reported to bind. We found that less than 40% of non-binding SLiMs have a selectivity value > 5, whereas over 80% of binding SLiMs have a selectivity value greater than 5. Higher selectivity values correspond to a higher specificity of interaction (Figure 2B). These results demonstrate that predicting domain binding to SLiMs based on motifs from peptide library experiments is effective.

### Global Conservation Analysis of SLiMs Recognized by SH2, SH3, PDZ and S/T Kinase Domains

**Figure 3:**
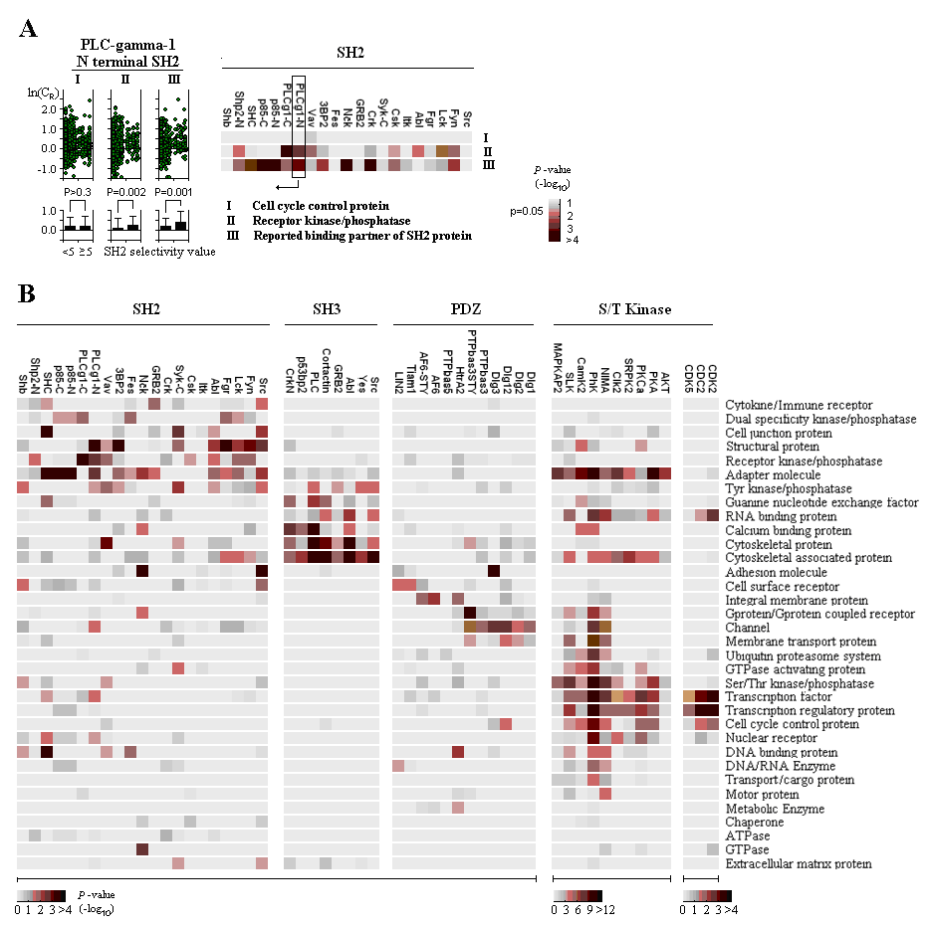
**Conservation analysis of SLiMs recognized by SH2, SH3, PDZ and S/T kinase domains in different protein functional classes**. (A) Conservation analysis of potential SH2 binding Tyr-SLiMs in cell cycle control proteins, receptor kinases/phosphatases and reported SH2 binding partners. The PLC-gamma-1 N terminal SH2 domain is shown as an example (left panel). The x-axis represents the selectivity of the PLC-gamma-1 N terminal SH2 domain, and the y-axis indicates the logarithm of C_R_. The Mann-Whitney test was performed to calculate the significance of the increase of conservation between SH2 non-selected (selectivity value < 5) and selected (selectivity value ≥5) Tyr-SLiMs. A color-coded map of p-values is shown on the right. (B) Conservation analysis of SLiMs recognized by SH2, SH3, PDZ and S/T kinase domains in different protein functional groups. Color-coded maps of p-values are shown below.

Using the PLC-γ1 N-terminal SH2 domain as a model to study the relationship between conservation and function of SLiMs, we found that Tyr-SLiMs predicted to bind to the PLC-γ1 N-terminal SH2 domain (selectivity value ≥ 5.0) have significantly higher ln(C_R_) scores, compared to Tyr-SLiMs in PLC-γ1 binding proteins (Mann-Whitney test, p = 0.001; Fig. 3A, left panel, III) and receptor kinase/phosphatase proteins (p = 0.002; Fig. 3A, left panel, II) not predicted to bind to the N-terminal SH2 domain (selectivity value < 5.0). No significant increase in ln(C_R_) score was observed for SH2-recognized (SH2 selectivity ≥5.0) Tyr-SLiMs in cell cycle control proteins (p > 0.3, Fig. 3A, left panel, I); importantly, cell cycle control proteins are rarely reported to bind to SH2 proteins.

Taking into consideration all binding partners, we found that for 20 of the 21 SH2 motifs, Tyr-SLiMs recognized by SH2 domains (selectivity value ≥5) have a higher average ln(C_R_) score than those not recognized by SH2 domains (selectivity value < 5); 11 of these are statistically significant (p < 0.05). In the receptor kinase and phosphatase group, 8 cases showed a significant increase in ln(C_R_) score. However, no significant increase in ln(C_R_) score was observed in the cell cycle control protein group (Figure 3A, right panel).

We then systematically examined the conservation of SLiMs recognized by SH2, SH3, PDZ and S/T Kinase domains (selectivity value ≥5) in representative protein functional classes taken from the Hprd database (Figure 3B). Those functional groups that show significant increase of conservation highly correlated with those that frequently interact with respective domains (functional classes frequently reported to interact with each domain were listed in Table 2). We observed that SH2-recognized SLiMs (Figure 3B, first panel) are most highly conserved in receptor kinases/phosphatases, adaptor molecules, tyrosine kinases/phosphatases and structural proteins; conservation was occasionally found in cytokine/immune receptors, cell junction proteins and cytoskeletal-associated proteins. Most other functional protein classes had little conservation signal. This result correlated well with those protein functional groups frequently interact with SH2 proteins as listed in Table 2. There are also some sporadic signals such as Nck in adhesion molecules and GTPase, Vav in cytoskeletal proteins and SHC in DNA binding proteins, suggesting they may interact with proteins in those functional groups.

**Table 2:** Molecular functional classes frequently reported to interact with SH2, SH3 or PDZ domains, or to be phosphorylated by S/T kinases

**Domain**	**Molecular function**	Binding ratio*
SH2	Receptor kinase/phosphatase	0.53
	Tyrosine kinase/phosphatase	0.51
	Cytokine/Immune receptor	0.36
	Adapter molecule	0.20
	Cell surface receptor	0.14
SH3	Tyrosinekinase/phosphatase	0.32
	Adapter molecule	0.18
	Guanine nucleotide exchange factor	0.12
	Cytoskeletal protein	0.11
	GTPase activating protein	0.11
PDZ	Channel	0.214
	Adhesion molecule	0.075
	Cell surface receptor	0.052

**Kinase**		**Phospho ratio**^#^

S/T Kinase	Serine/threonine kinase/phosphatase	0.00442
	Cell cycle control protein	0.00397
	RNA-binding protein	0.00334
	Transcription factor	0.00320
	Adapter molecule	0.00296
	Structural protein	0.00259
	Transcription regulatory protein	0.00255

* The binding ratio is calculated as the percentage of proteins that interact with proteins containing SH2, SH3, or PDZ domains.

# The phosphorylation ratio is calculated as the ratio of serine residues that are phosphorylated.

For SH3-recognized SLiMs (Figure 3B, second panel), conservation was strongest in cytoskeletal and cytoskeletal-associated proteins. calcium binding proteins, RNA binding proteins, tyr-kinases/phosphatases and guanine nucleotide exchange factors also had strong conservation signals. The conservation signal was almost absent in other functional classes. This is largely consistent with those frequently reported SH3 interacting protein groups (Table 2).

Consistent with biochemical evidences that PDZ domains frequently interact with membrane proteins, we found that PDZ domain-recognized SLiMs (Figure 3B, third panel) are specifically conserved in membrane proteins including channels, integral membrane proteins, cell surface receptors, G protein/G protein coupled receptors and membrane transport proteins. The frequent interacting partners of PDZ domain containing proteins are channels, adhesion molecules and cell surface receptors (Table 2). Our results suggest that those membrane proteins such as integral membrane proteins were probably less well studied but nevertheless play an important role in interaction with PDZ domain.

As shown in Figure 3B, fourth panel, the proteins containing SLiMs recognized by S/T kinases in the basophilic group (basophilic S/T kinases in this study included AKT, PKA, PKC, SRPK2, Clk2, NIMA, PhK, CamK2, SLK and MAPKAPK2) seem to be involved in a wider variety of cellular functions than proteins with SLiMs recognized by SH2, SH3 and PDZ domains. S/T kinase domain-recognized SLiMs were conserved in proteins involved in signal transduction (adaptor proteins and Ser/Thr kinase/phosphatases), in cytoskeletal-associated proteins, in proteins related to transcription and cell-cycle control, and also in some membrane proteins. However, the proteins containing conserved SLiMs recognized by proline-dependent Ser/Thr kinases (including CDK2, CDC2 and CDK5) were more specifically involved in transcription and cell-cycle control, with almost no conservation signal from other functional categories. The conservation pattern of SLiMs recognized by S/T kinases is highly consistent with protein functional groups with high serine phosphorylation ratio (Table 2).

Remarkably, most functional classes of proteins with a significant conservation signal were highly specific for the signal within one group of domains, but not in other groups. For example, receptor kinase/phosphatase group show conservation signal only in SH2 domain group and transcription factors only in Ser/Thr kinase domain group (Figure 3B) Nevertheless, a few protein functional classes exhibited a significant conservation signal in multiple groups of domains, such as adaptor molecules and cytoskeletal-associated protein groups; this corresponds to the fact that these proteins participate in multiple signaling pathways involving interactions with more than one domain.

**Figure 4:**
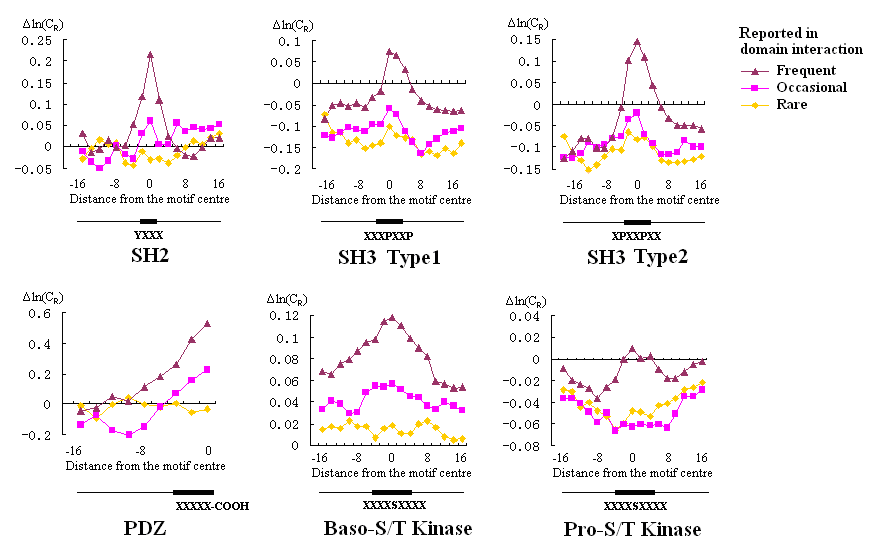
**Averaged conservation profiles of functional classes that are frequent, occasional or rare interaction partners of specific domains**. The plots show the change in ln(C_R_) between sequences containing SLiMs with high selectivity values and those containing SLiMs with low selectivity values for specific domains. The approximate SLiM regions are indicated with black boxes. Domain-recognized SLiMs are most conserved in protein functional classes that frequently interact with a specific domain.

In order to further examine the specificity of the conservation signal, we calculated the conservation profile of SLiMs in each protein functional class by calculating the difference in ln(C_R_) score between SLiMs with high selectivity and those with low selectivity. We also compared functional classes that are frequent, occasional or rare interaction partners for each domain by setting thresholds for the percentage of proteins in the functional class that either interact with or become phosphorylated by proteins containing that domain (Refer to Additional File 1 for detail. Frequent interaction partners for each domain were listed in Table 2). As expected, the conservation signal was highest in functional classes of proteins that are frequently reported to interact with a specific domain, and the signal progressively decreased for functional protein classes that are reported to interact occasionally or rarely with binding partners (Figure 4). Conservation profiles calculated as the change in ln(C_R_) score between SLiMs with upper-medium to medium selectivity values and SLiMs with low selectivity values showed similar trends, but were less significant (Figure S2, S3). In the above experiments, SLiMs for SH2 domains, PDZ domains or S/T Kinases with selectivity values of < 5, 5–10, 10–15, and > 15 were assigned to the categories of low, medium, upper medium and high selectivity, respectively; SLiMs with SH3 selectivity values of < 3, 3–6, 6–9, and > 9 were assigned to the categories of low, medium, upper medium and high selectivity, respectively.

### Conservation of SLiMs in Sub-Cellular Localization and in Multi-Domain Signaling

Using SH2 domain-interacting SLiMs as a model, we applied our method of conservation analysis to study additional aspects of SLiM conservation. Specifically, we investigated the conservation of SLiMs in proteins that interact with two different protein domains in a signaling pathway, and we studied the relationship between conservation of SLiMs and sub-cellular localization.

**Figure 5:**
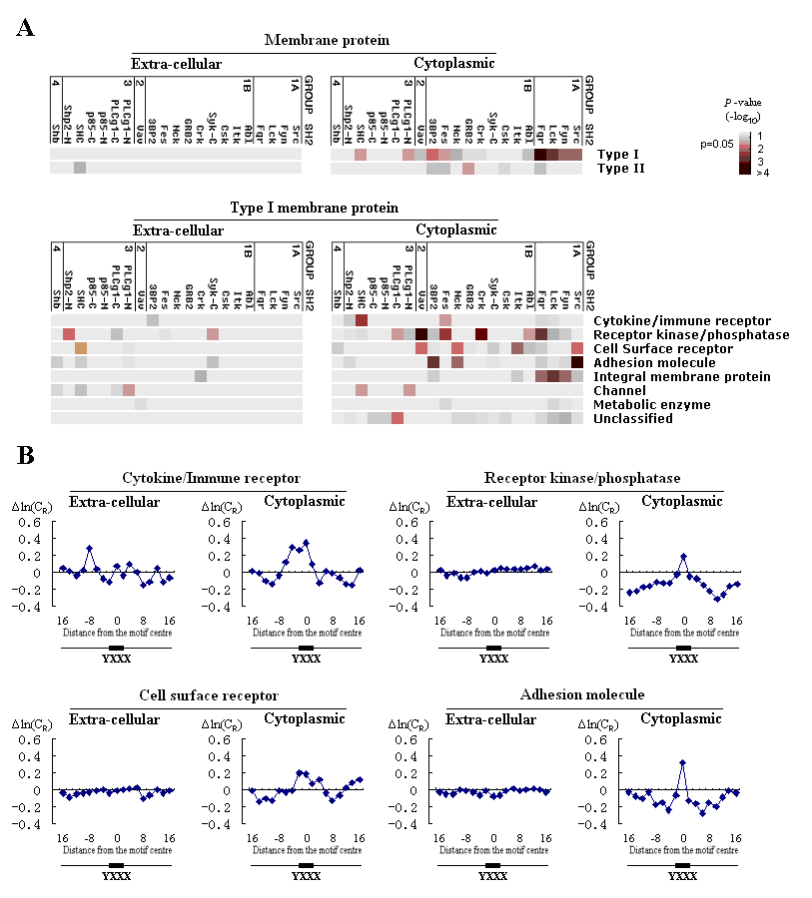
**SH2-recognized Tyr-SLiMs in membrane proteins are conserved in the cytoplasmic but not the extra-cellular region**. (A) The conservation signal in SH2-recognized Tyr-SLiMs is stronger in the cytoplasmic region than in the extra-cellular region in both Type I and II membrane proteins, especially in Type I membrane proteins from specific functional classes (including cytokine/immune receptors, receptor kinases/phosphatases, cell surface receptors, and adhesion molecules). (B) Examples of conservation profiles that compare extra-cellular regions to cytoplasmic regions.

**Figure 6:**
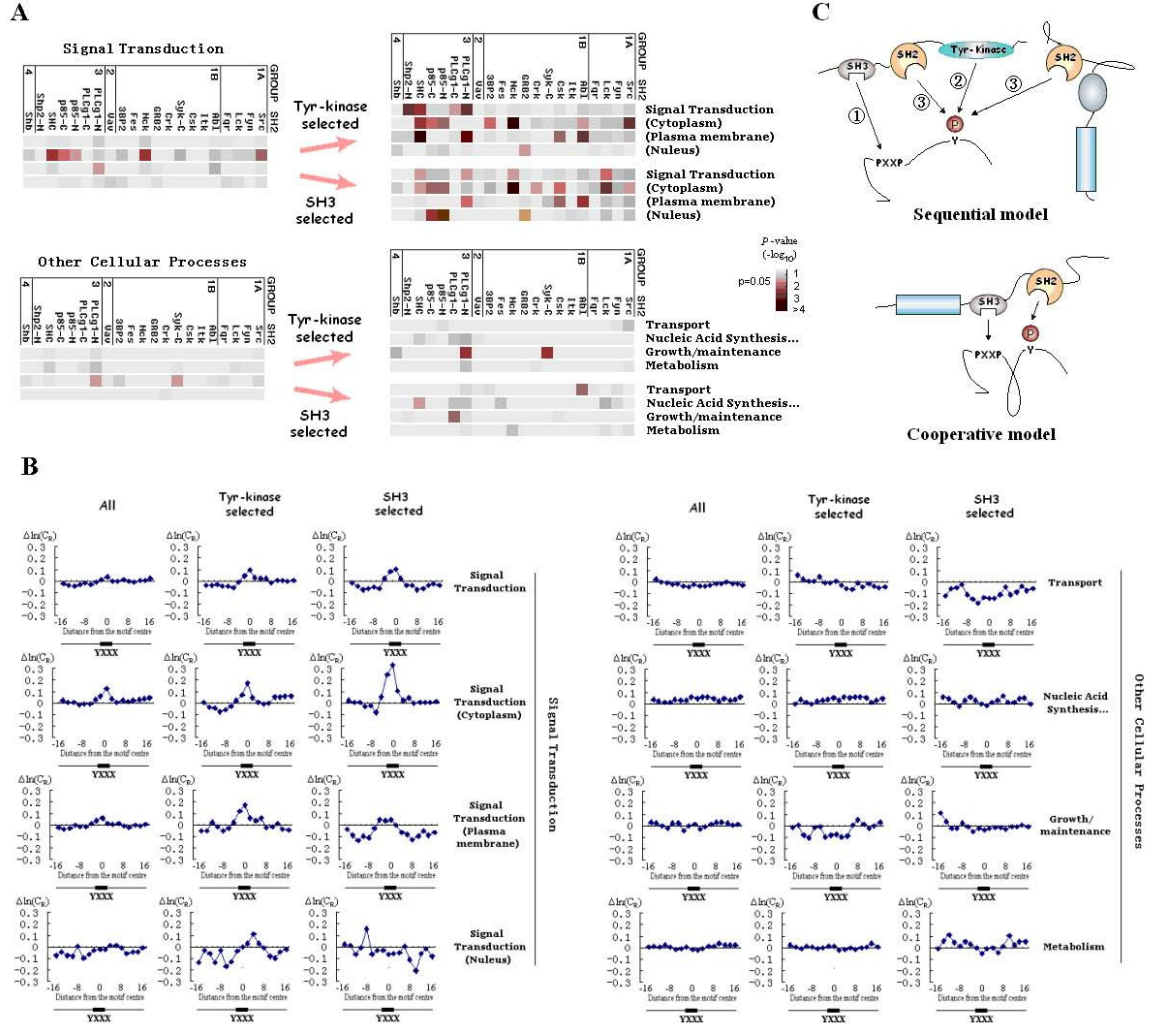
**Conservation analysis of Tyr-SLiMs after tyrosine kinase and SH3 domain selection**. (A) Comparison of relative conservation of SH2-recognized Tyr-SLiMs before and after Tyr-kinase and SH3 domain selections. (B) Conservation profile before and after Tyr-kinase and SH3 domain selections. (C) Schematic illustrations of the sequential model and the cooperative model to describe the coupling between SH2 and SH3 domains.

Consistent with the observation that SH2-mediated signaling mainly occurs in the cytoplasm, we found a conservation signal for SH2-recognized SLiMs in cytoplasmic but not extra-cellular regions in both Type I and II membrane proteins (Figure 5A). (For Type I membrane proteins, the cytoplasmic side is C-terminal, while for Type II membrane proteins it is N-terminal.) Since the majority of membrane proteins are Type I, we further classified this group by protein function. The conservation signal is strongest for SLiMs on the cytoplasmic side of receptor kinases/phosphatases, cell surface receptors, cytokine/immune receptors and adhesion molecules, and weaker for SLiMs in channels and metabolic enzymes (Figure 5B). SH2-domain binding is dependent on tyrosine phosphorylation, which is catalyzed by Tyr kinases. Accordingly, SLiMs recognized by Tyr kinases should be more likely to interact with SH2 domains. We found that SH2-recognized SLiMs that were selected for based on the presence of a common tyrosine kinase motif (containing E/D up to four amino acids from the tyrosine on the N-terminal side) are more conserved than those without this selection (Figure 6A).

On the other hand, many tyrosine kinases (including the well-known Src family kinases) and adaptor molecules have both SH2 and SH3 domains, and it has been suggested that proteins containing multiple SH3 binding sites are more likely to be tyrosine phosphorylated and bind to SH2 domains as supported by biochemical studies [10,11]. Consistent with this reasoning, SH2-recognized Tyr-SLiMs in signal transduction proteins that have more than ten PXXP SH3 binding motifs are significantly more conserved than SLiMs without this selection (Figure 6A). However, this trend is not observed in SLiMs in functional classes other than signal transduction protein class (Figure 6A), which agrees well with the fact that most SH2-binding proteins are signal transduction proteins.

We further divided signal transduction groups into subgroups according to sub-cellular localization. Under selections for both the kinase motif and SH3 binding motifs, a high level of SLiM conservation was most manifest in signal transduction proteins localized to the cytoplasm or plasma membrane, but conversation of SLiMs was weaker for those proteins localized to the nucleus (Figure 6A). This is consistent with biochemical evidence that tyrosine phosphorylation occurs mainly in the cytoplasm and plasma membrane (the ratios of proteins that bind to SH2-containing proteins in the cytoplasm, plasma membrane and nucleus are 16.1%, 11.4% and 4.7% respectively, according to Hprd). Conservation profiles for different functional classes of proteins with or without SH3 and Tyr-Kinase domain selection are shown in Figure 6B.

These findings support the hypothesis that tyrosine kinases and SH3 domains are frequently coupled to SH2 domain signaling. The coupling between a tyrosine kinase and SH2 domains is expected, since an SH2 domain can only bind to a Tyr-SLiM after the tyrosine residue has been phosphorylated by a Tyr-kinase. However, the coupling between SH2 and SH3 domains might be less direct. Either a sequential model or a cooperative model, depending on whether the target tyrosine residue is phosphorylated before the interaction, may be used to explain the coupling between SH2 and SH3 domains (Figure 6C). In the sequential model, PXXP motifs recruit SH3 domain containing Tyr-kinases, which in turn phosphorylate the tyrosine residues in the target protein. The pYXXX motif can then recruit an SH2 domain (Figure 6C, upper panel). In the cooperative model, the SH2 and SH3 domains in a single kinase or adaptor molecule bind to the pYXXX motif and the PXXP motif, respectively, to increase the strength of the interaction (Figure 6C, lower panel). Both of these models may explain the coupling between SH2 and SH3 domains. Early in tyrosine phosphorylation-mediated signal transduction, most tyrosine residues are not phosphorylated, so the sequential model may prevail. However, after more tyrosine residues in signaling proteins become phosphorylated, the cooperative model may become increasingly relevant.

## Discussion

Protein-protein interactions mediated by SLiMs have a widespread influence on cellular functions[12,13]. In this study, we examined these interactions by combining scoring matrixes derived from peptide library and conservation analysis. We detected signals of evolutionary conservation in SLiMs in proteins from functional classes that are known to participate in the signal transduction of a specific protein domain. Further, our analysis of membrane proteins indicated that only the cytoplasmic side is involved in SH2 signaling in both Type I and II membrane proteins. Our results also suggest that tyrosine kinase and SH3 domains are coupled with SH2 domain signaling in signal transduction proteins.

It was recently reported that several bacterially secreted cytotoxins contain multiple repeated Tyr-SLiMs with high affinity for both tyrosine kinases and SH2 domains [14-17]. Many of these cytotoxins are phosphorylated upon entry into host cells and bind to a variety of SH2 proteins. For example, the CagA protein secreted by *Helicobacter pylori *can be phosphorylated by Src and associates with Shp2 [18] and Csk [18] SH2 domains, which is essential for cellular changes induced by the bacteria. The strong cellular response initiated by these SH2 binding Tyr-SLiMs further supports our assumption that SLiMs are under continuous evolutionary selection to preserve functional sites and eliminate harmful mutations. Recent work on the negative selection of SH3 domain-recognized sequences [19] also suggests that SLiMs may undergo strong evolutionary selection.

While most protein functional classes with strong conservation signal are known to be involved in the signaling of respective domains, there are a few exceptions, which may represent undiscovered but functional binding sites. For example, Although less than 3% structural and cytoskeletal proteins have been recorded to bind to SH2 proteins, their Tyr-SLiMs selected by SH2 domains had significantly increased C_R _scores. It has been reported that alpha-Tubulin, a cytoskelatal protein, binds to the Fyn SH2 domain [20], and that the intermediate filaments of the cytokeratin type are reported to undergo tyrosine phosphorylation [21]. In the latter case, further evaluation is necessary to determine whether the phosphorylation leads to SH2 binding.

Another interesting observation is that DNA binding proteins also have conservation signal in their potential SH2 binding sites. Although tyrosine phosphorylation is generally believed to be less common in the nucleus, more and more evidences for the tyrosine phosphorylation of DNA binding proteins are reported as in the case of KRC DNA binding protein [22], estrogen receptor [23], TFII-I [24] and more examples provided in [25]. Since many SH2-containing proteins were reported to enter nucleus such as Fes [26], SHC [27], Nck [28] and Vav [29]. SH2 domains may mediate functional interactions with DNA binding proteins. Similar to SH2 domain, we observed that DNA binding proteins also have conservation signal in potential PDZ binding sites. Although most reported interactions mediated by PDZ domains are restricted to membrane proteins, proteins that contain PDZ domain (for example, LIM-kinase 1 [30] and Par3 [31]) were reported to enter nucleus suggesting they may mediate protein-protein interactions in the nucleus. Whether these observations represent a new trend of research is worth investigation.

Although our results from conservation analysis correlated well with biochemical data in general, our method is still prone to error. First, our motif prediction is based on *in vitro *peptide scanning techniques, which may be biased due to differences between *in vitro *and *in vivo *conditions. Second, we assumed that each position of the SLiM contributed equally to binding, and only SLiMs that were conserved at each position were assumed to be conserved. To improve this method in the future, different weights could be assigned to each position, and amino acid similarity could be considered. Finally, evolutionary conservation can only provide indirect clues regarding function. For example, some SLiMs may only be important for a few species, and these would not have been detected in our analysis.

Our results indicate that the conservation pattern of SLiMs recognized by SH2, SH3, PDZ, and S/T kinase domains highly correlates with the function of these domains. As motifs recognized by other domains are better defined, conservation analysis will be able to provide valuable clues as to their functional roles, as well as possible preferences for their sub-cellular localization or for their coupling with other domains and even structural implications. For example, in a recently published paper [32], the authors show that SLiMs are more likely to be conserved in disordered protein regions. Recently, peptide array based technology has been developed and is becoming increasingly available [33,34]. New technologies are expected to make motif discoveries easier and potentially more accurate. Currently, many of the motifs discovered are only defined as regular expressions, which usually provide less information than those motifs defined from the result of peptide library screening. Nevertheless, it should be possible to retrieve useful information from those less well-defined motifs using more sophisticated algorithms in the future.

## Conclusion

This study systematically studied the evolutionary conservation of SLiMs recognized by SH2, SH3, PDZ and S/T Kinase domains which reflected the interactome of these domains. Specifically, SLiMs within certain protein functional groups that are frequently involved in the interaction with that domain are significantly more conserved than those SLiMs within other groups. Study of manually extracted SH2 interaction sites in 11 most studied receptor tyrosine kinases provided experimental evidence that Tyr-SLiMs reported to interact with SH2 are significantly more conserved than those that do not. Furthermore, by analysis of SLiMs in membrane proteins and under selection of two different domains, we show that this conservation analysis can also provide useful information about the sub-cellular localization of the interaction and domain coupling.

## Methods

### Selection and Classification of Human Protein Sequence Data

We selected 7,248 human proteins for our protein functional classification analysis and 8,682 proteins for our cellular process classification analysis, using the following criteria: (1) The protein had SwissProt annotated sequence; (2) The protein had a molecular function or cellular process annotated by the Human protein reference database (Hprd) [35]; (3) The molecular function or cellular process of the protein was within 34 well-represented functional classes of proteins in Hprd.

Human protein sequence data are from the SwissProt database, downloaded from  in November 2005. Protein-protein interactions, and classifications for protein molecular functions, biological processes and sub-cellular localizations are from the Hprd dataset [35]. This is a non-redundant manually-curated protein database, and data was downloaded in November 2005 from . Phosphorylated sites were obtained from the Phospho.ELM database [36] provided by Francesca Diella in December 2005. We excluded several sequence regions unlikely to contain SLiMs (globular domains, coiled-coils, collagen regions and signal peptides, as annotated in SwissProt), because no more than 15% of known SLiMs [12,37,38] occur in these regions.

### Selection of Homologous Proteins

Using human protein sequences selected as described above, we did pair-wise local alignments generated by BLAST [39] against 12 higher eukaryotic species (*Canis familiaris, Bos taurus, Mus musculus, Rattus norvegicus, Gallus gallus, Xenopus tropicalis, Tetraodon nigroviridis, Danio rerio, Strongylocentrotus purpuratus, Drosophila melanogaster, Apis mellifera*, and *Caenorhabditis elegans*) to obtain homologous sequences for the respective human proteins. Species were selected according to their unique evolutionary positions (four mammals, four non-mammal vertebrates and four invertebrates) and sequence availability in the RefSeq database [40]. Sequence data for all non-human species were from the RefSeq database downloaded from  in June 2006 except *Tetraodon nigroviridis*, which was from the NCBI Entrez non-redundant protein sequence database downloaded from  in June 2006. We applied two cutoff levels to avoid inclusion of insignificant hits: a score cutoff of 50 bits, and an overlap cutoff of 50%, as applied in Inparanoid [41]. If more than one homologous sequence was obtained from a single species, the one with the lowest E-value was selected. Unlike Inparanoid [41] or COG (Cluster of Orthologous Groups) [42], which consider all species as equal entries, we compared sequences of all other species to those of human, because most biochemical data we used including protein interaction data and protein classification data were from human. Therefore, we only considered the best hit from non-human species as homologous to the human query protein, but not necessarily mutually best matches between human and non-human species or non-human species themselves. We have not removed low complexity regions because SLiMs frequently occur within them.

### Calculation of the Conservation Score of SLiMs

SLiM occurrences were defined based on invariant features for each domain as listed in Table 1 (except Thr-SLiMs were not included in the analysis for Ser/Thr kinases domains because only peptide library mapped motifs for Ser-SLiMs were available). All occurrences in the proteins that matched these invariant features were included in the analysis. For example, all sequences with the pattern YXXX were selected. For a particular protein sequence, we assumed that the sequence identity rate between a reference species (human in this study) and a species i is p_(*i*) _(equal to the number of identical sites divided by the total number of sites aligned. In cases where gaps occur in the alignment sequence of species i, the number of gaps was subtracted from the number of sites aligned as the final alignment length), and that the SLiM under study is n amino acids in length (in cases where the SLiM is at the terminus of a protein and is only partially available, the available length was considered). If the SLiM is under the same evolutionary selectivity as the full-length protein, then the probability that the SLiM is conserved between the two species should be:

P_1_(i) = p(i)^n^

The probability that the SLiM is unconserved should be:

P_2_(i) = 1- P_1_(i) = 1-p(i)^n^

The SLiM is considered unconserved if any gap occurs within its sequence alignments.

Here we define Relative Conservation (C_R_) between human and the i^th ^species as:

a. if the SLiM is conserved:

C_R_(i) = 1/P_1_(i) = 1/p(i)^n ^;

b. if the SLiM is unconserved:

C_R_(i) = P_2_(i) = 1-p(i)^n ^;

If C_R_(i) from k different species are [C_R_(1), C_R_(2), C_R_(3),..., C_R_(k)], then C_R _of the SLiM among different species is calculated as:

$${\text{C}}_{\text{R}}=\text{k}\sqrt{\prod_{\text{i}=1}^{\text{k}}{\text{C}}_{\text{R}}(\text{i})}$$

A C_R _score greater than 1 indicates the SLiM is C_R _times more conserved than the average level of the protein. A score smaller than 1 indicates 1/C_R _times greater variability between species. Note that the number k may be different for different SLiMs according to the pair-wise Blast results.

This method may not be suitable for SLiMs longer than 10 amino acids, since it assumes that most residues in the SLiM could influence the interaction. This may not be the case in longer sequences where only a small subset of the residues is critical to binding. This method was first developed in our lab and has demonstrated its effectiveness in another research[32] where SLiMs were found to be more conserved in disordered protein regions.

### Definition of Domain Selectivity

For a putative SLiM, the selectivity value for domains were calculated as the product of enrichment values from peptide library experiments [43,44]. For example, to calculate the Src SH2 selectivity value of the SLiM YENF, we found the enrichment values for E(Y+1) and N(Y+2) for Src SH2 (Table 3) are 2.5 and 2.4, respectively. No enrichment value for F(Y+3) was found (thus Y+3 does not contribute to the final value) and the selectivity value is the product of the two enrichment values (2.5 × 2.4 = 6.0). The enrichment values for SH3 domain recognized motifs were assigned based on amino acid sequence of peptides expressed by SH3-binding phage clones [45].

Please see Additional File 1 for more methods.

**Table 3:** Enrichment values for the Src SH2 domain

**PY+1**	**pY+2**	**pY+3**
E(2.5)	E(2.6)	I(3.6)
D(1.7)	N(2.4)	M(2.5)
T(1.7)	Y(2.0)	L(2.3)

## Competing interests

The authors declare that they have no competing interests.

## Authors' contributions

SR was involved in design and planning of the experiments, has done the computational analysis and drafted the manuscript. GY, YH and YW were involved in carrying out experiments and computational analysis. YL was involved in planning of the experiments. ZC was involved in design and planning of the experiments, drafted the manuscript and headed the project. All authors have read and approved the final manuscript.

## Supplementary Material

Additional file 1 **Additional methods and results.** This additional file presents additional methods and results related to this article. Click here for file
