# Supplementary material for: The conservation pattern of short linear motifs is highly correlated with the function of interacting protein domains
Source: BMC Genomics. 2008 Oct 1;9:452. doi: 10.1186/1471-2164-9-452 (PMC2576256; doi:10.1186/1471-2164-9-452)
Supplement: Additional file 1 — Additional methods and results. This additional file presents additional methods and results related to this article. [file 1471-2164-9-452-S1.pdf]

ADDITIONAL METHODS

Domain and kinase selectivity data (motifs)

21 SH2-binding motifs analyzed were Src, Fyn, Lck, Fgr, Abl, Crk, Nck, p85N, p85C, PLCγ1N, PLCγ1C and SHPTP2N (Songyang, et al., 1993); Csk, 3BP2, Fes, GRB2, SHC, Syk and Vav (Songyang, et al., 1994); Shb (Karlsson, et al., 1995); and Itk (Bunnell, et al., 2000). 8 SH3 motifs are Src, Yes, Abl, Cortactin, p53bp2, PLCγ, Crk and Grb2 (Sparks, et al., 1996); 12 PDZ motifs are Dlg-1, Dlg-2, Dlg-1/2, Dlg-3-STY, PTPbas-3, PTPbas-3-STY, PTPbas-5, p55, Tiam-1, AF-6, AF-6-STY (Songyang, et al., 1997), HtrA2 (Martins, et al., 2003); 13 Ser/Thr Kinase motifs are NIMA, PhK, CamK, CDK5 (Songyang, et al., 1996), CDC2, CDK2, PKA, SLK1 (Songyang, et al., 1994), AKT (Yaffe, et al., 2001), PKC (Nishikawa, et al., 2000), SRPK2 (Wang, et al., 1998), MAPKAPK-2 (Manke, et al., 2005) and CLK2 (Nikolakaki, et al., 2002); Src kinase and common Tyr-kinase motifs are from (Songyang, et al., 1995).

Calculation of statistical significance

To explore the functional importance of domain recognized SLiMs, we compared SLiMs with domain selectivity values equal to or greater than 5 to those less than 5 in each molecular functional group. A selectivity value greater than 5 generally means that 3 strongly selected sites (enrichment value >1.7) or more number of weakly selected sites which is often adequate for effective interaction. We applied the Mann-Whitney test to quantitatively measure the significance of differences in relative conservation score. We randomly picked a standard number (2000 for SH2 and SH3; 500 for PDZ and 6000 for S/T Kinases) of SLiMs from each molecular functional group for statistical analysis. In those groups that do not have enough number of SLiMs, all the SLiMs were selected. The sampling process is repeated 1000 times and the final p-value was obtained from the average Z score of the 1000 trials.

Similar sampling is performed in the part of SH2 domains coupling with Tyr-Kinases and SH3 domains where proteins are grouped according to cellular processes. Since these groups were generally large, we have made three independent tests to enhance the sensitivity. Coservation scores of SLiMs with medium (5 to 10), upper medium (10 to 15) and high (≥15) SH2 selectivity values, were each compared with those of low SH2 selectivity values (<5), and the lowest of the three *p*-values was chosen. It should be mentioned that in the latter case similar trends can be observed using SH2 selectivity 5 as a single cut-off for statistical tests (Figure S6).

Definition of frequent, occasional and rare binding partner (substrate) group

For SH2, SH3 amd PDZ domains, frequent, occasional and rare binding partner groups are defined by setting thresholds of the percentage of proteins in the functional group that interact with proteins containing that domain according to Hprd data set (Peri, et al., 2004). For SH2 and SH3 domains the thresholds are >10%, 1-10% and <1% for frequent, occasional and rare binding partners respectively. PDZ domains are known to frequently bind to membrane proteins, so we set thresholds only within membrane molecular functional groups, >5% for frequent, <5% for occasional binding partners. Non-membrane molecular functional groups are classified as rare binding partners. For S/T Kinases, we set thresholds with the ratio of Serine phosphorylation according to PhosphoELM (Diella, et al., 2004) >2.5‰, 1.5-2.5‰, <1.5‰ for frequent, occasional and rare substrate groups respectively. In order to make the results more reliable, we excluded those protein functional groups that have fewer than 150 PXXP motifs for SH3 domains (31 functional groups remained), fewer than 100 proteins (=100 C-terminal SLiMs) for PDZ domains (22 functional groups remained) and fewer than 3000 Ser-SLiMs for S/T Kinase domains (24 functional groups remained). All 34 functional groups were included for SH2 domains.

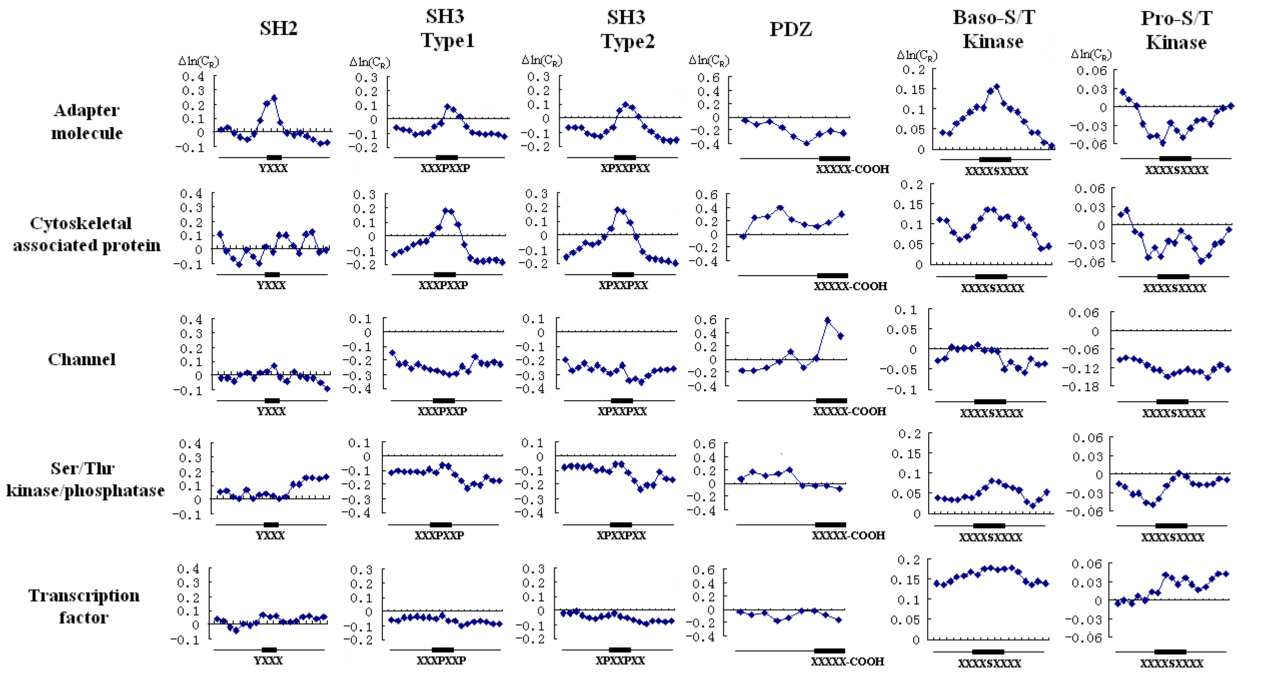

Figure S1 Examples of conservation profiles of SLiMs recognized by different domains in several molecular functional groups. Approximate SLiM region are indicated with black boxes.

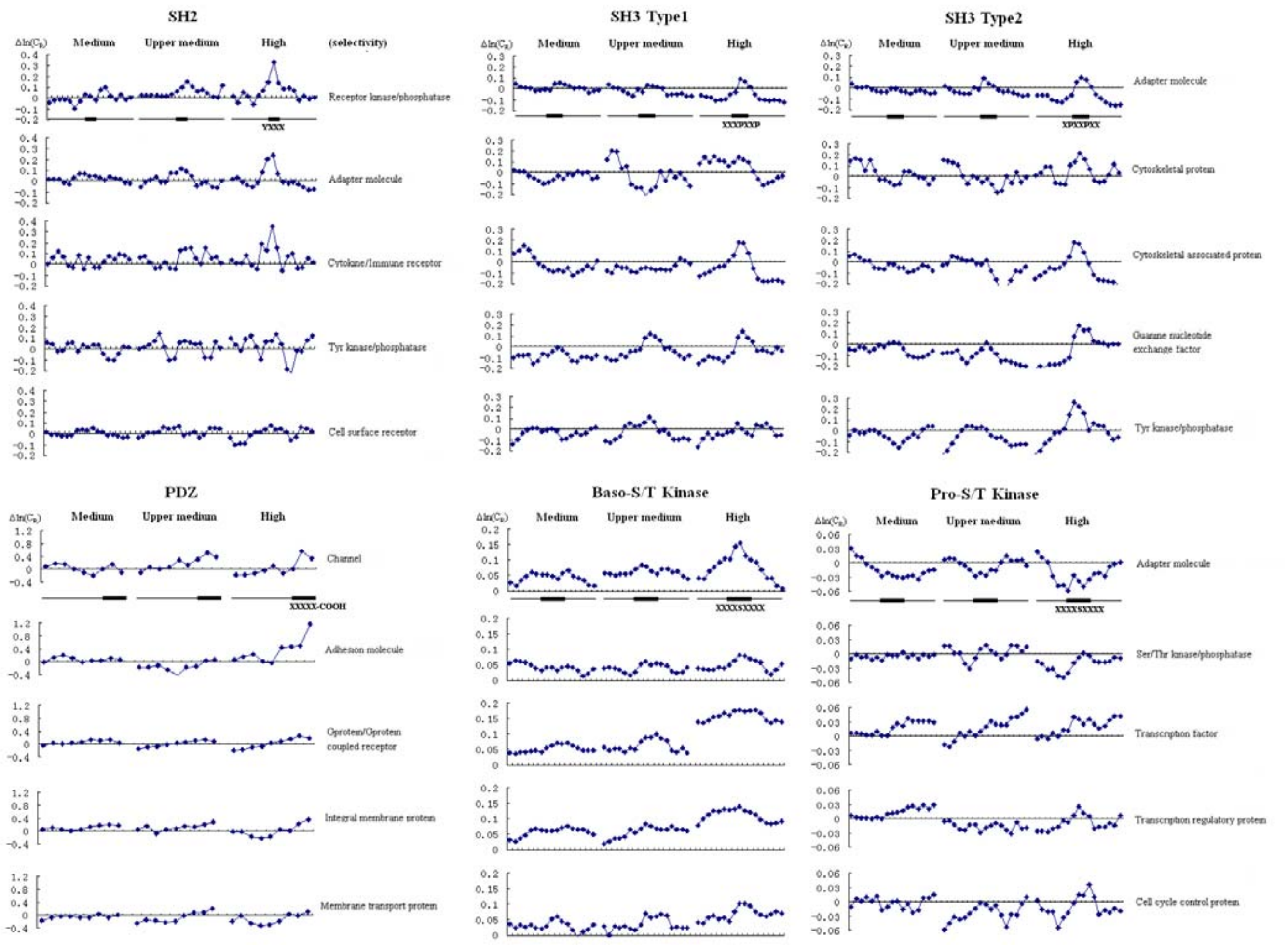

Figure S2. Examples of conservation profiles of SLiMs under different selectivity values. The plots show the Δln(C_R) between sequences containing SLiMs with High, Upper medium, medium selectivity values and those that containing SLiMs with low selectivity values.

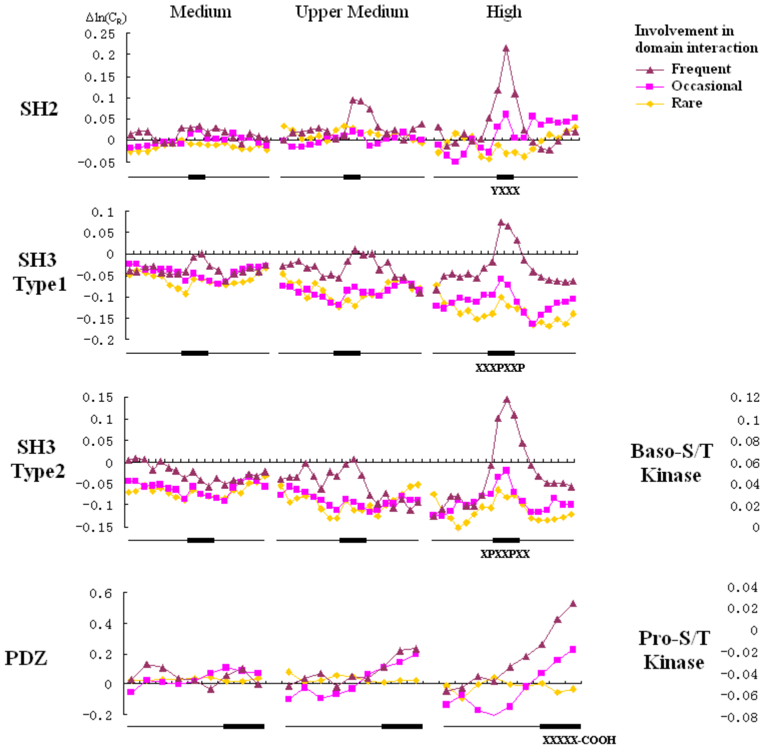

Figure S3 Averaged conservation profiles of SLiMs with medium, upper medium and high selectivity values for SH2, SH3 PDZ and S/T Kinase domains in functional groups that are frequent, occasional or rare interaction partners of each domain.

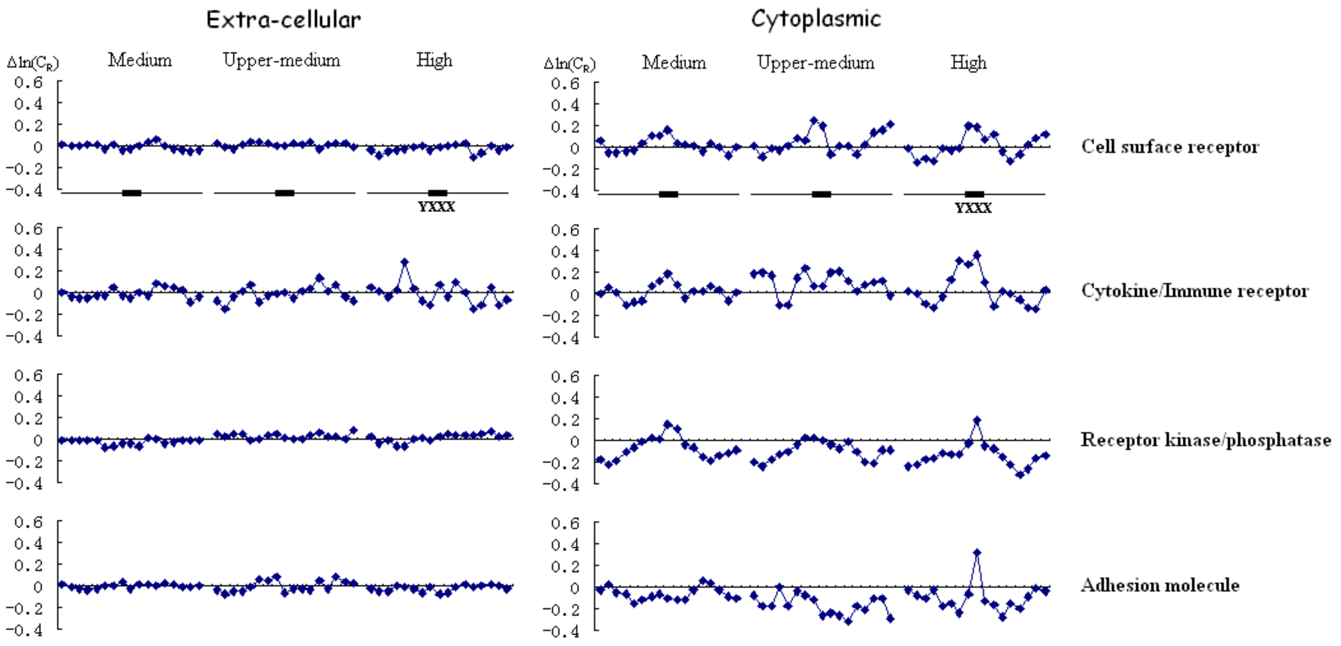

Figure S4 Examples of conservation profiles of Tyr-SLiMs with medium, upper medium and high selectivity values for SH2 domain in cytoplasmic or extracellular regions of Type I membrane proteins.

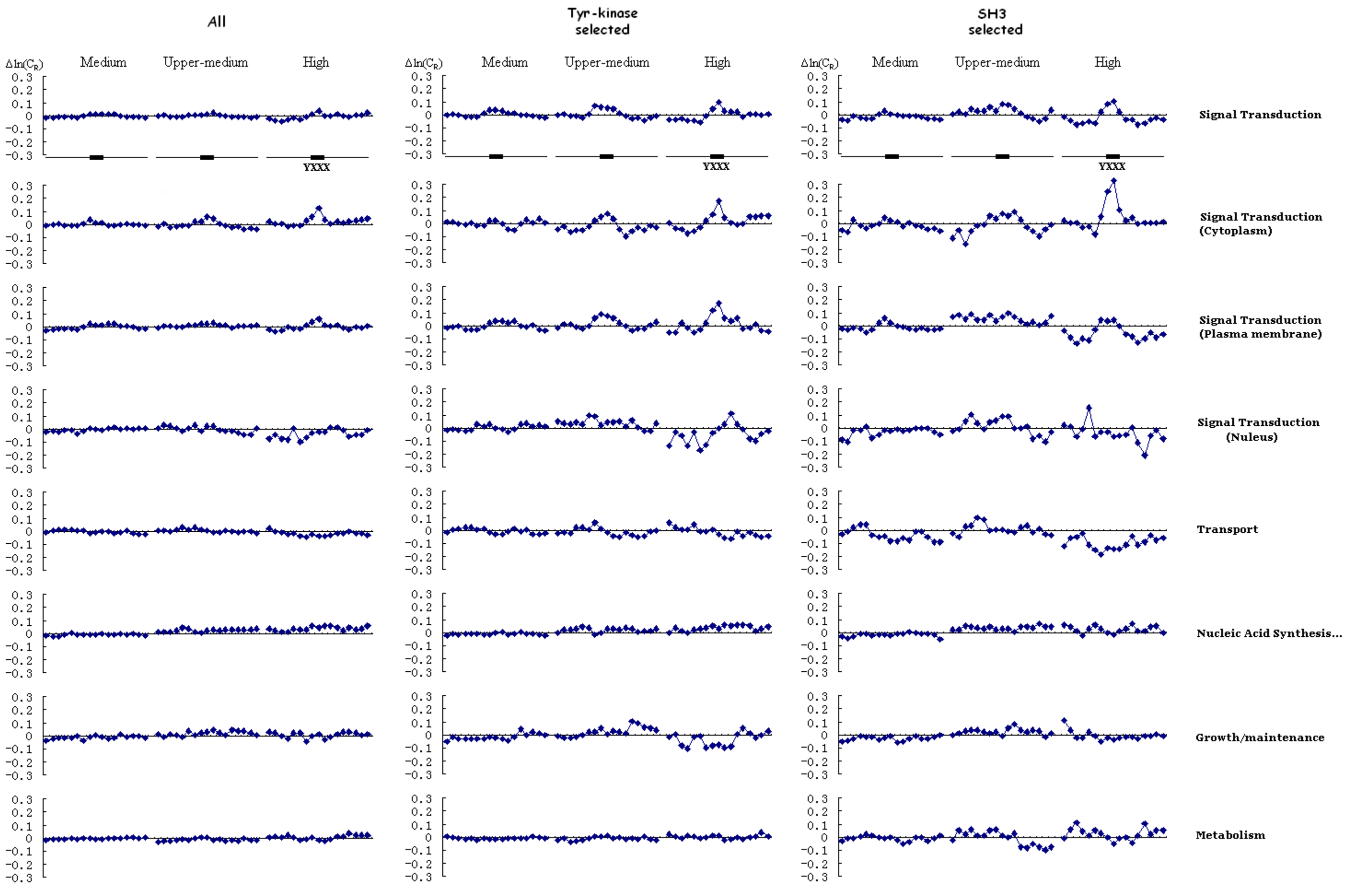

Figure S5 Examples of conservation profiles of Tyr-SLiMs with medium, upper medium and high selectivity values for SH2 domain before and after Tyr-kinase and SH3 domain selections.

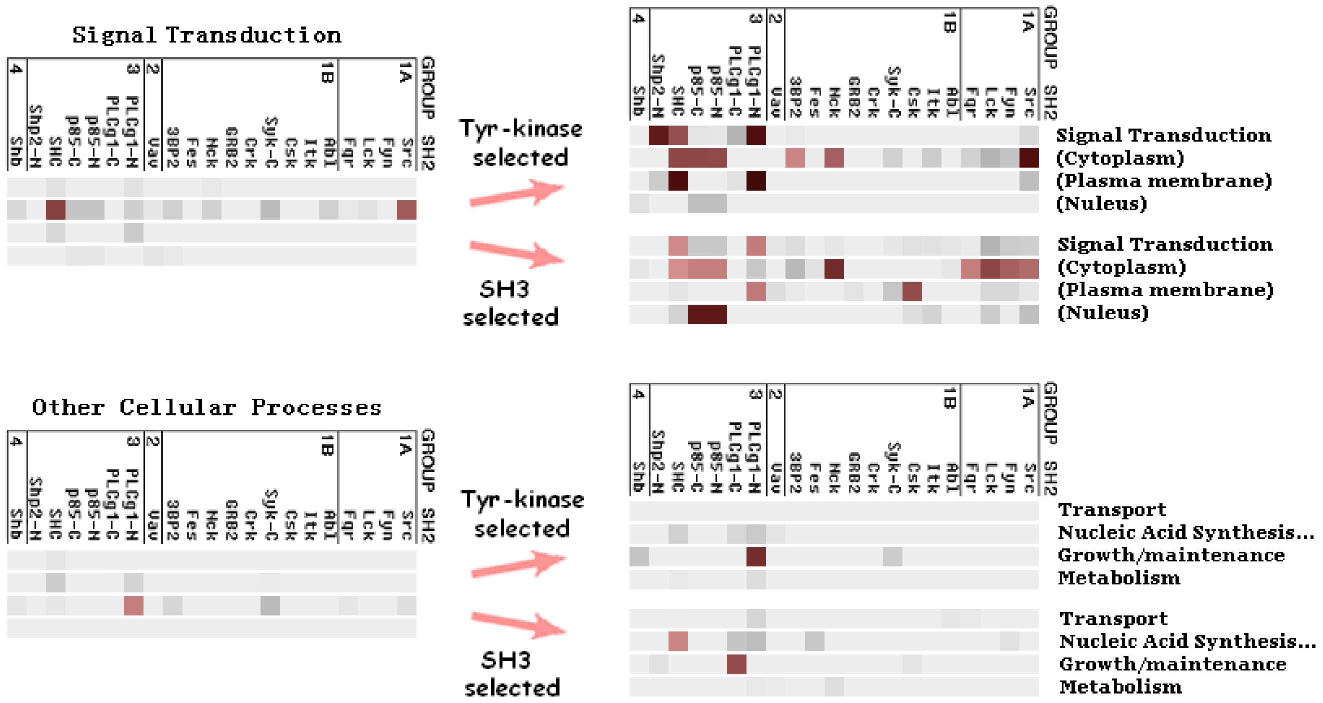

Figure S6

Conservation analysis of Tyr-SLiMs with a single cut-off selectivity value before and after tyrosine kinase and SH3 domain selections

Table S1 Reported SH2 binding sites in 11 most studied RTKs

Sites	ln(Cr)	Sequence	Reference
**EGFR**			
727	0.494	FGTVYKGLW	Shc (Schulze WX et. al. 2005)
915	0.763	GSKPYDGIP	Src (Stover DR. et. al. 1995)
944	0.763	TIDVYMIMV	p85 (Stover DR. et. al. 1995)
978	0.564	DPQRYLVIQ	Shp2 (Schulze WX et. al. 2005)
998	0.203	DSNFYRALM	Shc (Schulze WX et. al. 2005), Shp2 (Schulze WX et. al. 2005), Crk (Schulze WX et. al. 2005)
1016	0.004	DADEYLIPQ	PLCg1(Rotein et. al. 1992), Shp2 (Schulze WX et al. 2005)
1092	-0.239	PVPEYINQS	GRB2 (Batzer et. al. 1994, Schulze WX et. al. 2005)
1110	0.243	QNPVYHNQP	GRB2 (Okutani et. al. 1994, Schulze WX et. al. 2005)
1125	-0.742	RDPHYQDPH	GRB2 (Schulze WX et al. 2005)
1138	0.558	GNPEYLNTV	GRB2 (Schulze WX et al. 2005)
1197	-0.049	ENAEYLRVA	Shc (Sakaguchi et. al. 1998, Schulze WX et. al. 2005), PLCg1 (Chattopadhyay et. al. 1999)
**ERBB2**			
735	0.339	FGTVYKGIW	Shc (Schulze WX et. al. 2005)
952	0.548	TIDVYMIMV	p85 (Ram TG et. al. 1996)
1005	0.153	DSTFYRSLL	Shc (Schulze WX et. al. 2005)

1023	0.873	DAEEYLVPQ	Shp2 (Schulze WX et. al. 2005)
1139	0.472	PQPEYVNQP	GRB2 (Ricci A. et. al. 1995, Schulze WX et. al. 2005)
1196	-0.271	ENPEYLTPQ	Shc (Schulze WX et. al. 2005)
1222	0.234	DNLYYWDQD	Shc (Schulze WX et. al. 2005)
1248	0.969	ENPEYLGLD	Shc (Ricci A. et. al. 1995, Schulze WX et. al. 2005)
**FGFR**			
463	0.433	GVSEYELPE	Crk (Larsson, H et. al. 1999)
730	0.099	TNELYMMMR	PLCg1 (Mohammadi M et. al. 1991)
766	0.694	SNQEYLDLS	Shb (Cross MJ et. al. 2002), PLCg1 (Mohammadi M et. al. 1991)
**IGFIR**			
973	0.588	NGVLYASVN	Crk (Koval AP et. al. 1998) , Csk (Arbet-Engels C. et. al. 1999)
980	0.182	VNPEYFSAA	Crk (Koval AP et. al. 1998)
1346	0.583	ERQPYAHMN	Csk (Arbet-Engels C. et. al. 1999), p85 (Seely BL. et. al. 1995), Shp2 (Seely BL. et. al. 1995),
**IR**			
1185	1.045	TRDIYETDY	Shp2 (Kharitonenkov A et. al. 1995)
1361	0.255	EHIPYTHMN	Shp2 (Kharitonenkov A et. al. 1995), Csk (Arbet-Engels C. et. al. 1999), p85 (Van Horn DJ. et. al. 1994)
**KIT**			
568	0.926	NGNNYVYID	Lck (Krystal GW et. al. 1998), Shp2 (Kozlowski M. et. al. 1998)
570	0.926	NNYVYIDPT	Lck (Krystal GW et. al. 1998)
703	0.160	EAALYKNLL	Grb2 (Thommes K et. al. 1999)
721	0.312	STNEYMDMK	p85 (Herbst R et. al. 1995)
900	0.310	PAEMYDIMK	Crk (Lennartsson J et. al. 2003)
936	0.298	TNHIYSNLA	Grb2 (Thommes K et. al. 1999)
**MET**			
1313	0.559	PDPLYEVML	p85 (Maulik G. et. al. 2002)
1349	0.270	IGEHYVHVN	Shc (Pelicci G. et. al. 1995)
1356	0.270	VNATYVNVK	GRB2 (Ponzetto C. et. al. 1996), Shc (Pelicci G. et. al. 1995), Shp2 (Fixman ED. et. al. 1996), PLCg1 (Fixman ED. et. al. 1996)
**PDGFR**			
579	1.340	DGHEYIYVD	Src (Mori S et. al. 1993), Shc (Yokote K et. al. 1994)
581	1.340	HEYIYVDPM	Src (Mori S et. al. 1993)
716	0.243	SAELYSNAL	GRB2 (Amidsson AK et. al. 1994)
740	1.340	SDGGYMDMS	p85 (Panayotou G et. al. 1992), Shc (Yokote K et. al. 1994)
751	1.340	ESVDYVPML	p85 (Panayotou G et. al. 1992), Shc (Yokote K et. al. 1994)
771	0.243	ESSNYMAPY	Shc (Yokote K et. al. 1994)
1009	0.500	SSVLYTAVQ	Shp2 (Lechleider RJ et. al. 1993)
1021	1.340	GDNDYIIPL	PLCg1 (Ronnstrand L et. al. 1992)
**RET**			

981	-0.101	SEEMYRLML	Src (Encinas M et. al. 2004)
1015	1.306	KRRDYLDLA	PLCg1 (Borrello, M et. al. 1996)
1062	0.983	ENKLYGMSD	Shc (Asai N et. al. 1996)
1096	1.185	NDSVYANWM	GRB2 (Alberti, L et. al. 1998)
**TKRA**			
496	0.123	ENPQYFSDA	Shc (Obermeier A et. al. 1993)
680	1.420	YSTDYYRVG	Grb2 (MacDonald JI, et. al. 2000)
681	0.927	STDYYRVGG	Grb2 (MacDonald JI, et. al. 2000)
757	0.098	PPEVYAIMR	p85 (Obermeier A et. al. 1993)
791	0.094	APPVYLDVL	Abl (Yano H et. al. 2000), Grb2 (MacDonald JI, et. al. 2000)
**VEGFR2**			
801	0.646	LKTGYLSIV	PLCg1 (Cunningham SA. et. al. 1997)
1175	0.048	DGKDYIVLP	Shb (Holmqvist K. et. al. 2004), PLCg1 (Cunningham SA. et. al. 1997)
1214	0.266	PKFHYDNTA	Nck (Lamalice L et. al. 2006)

Supplemental references

Bunnell, S.C., Diehn, M., Yaffe, M.B., Findell, P.R., Cantley, L.C. and Berg, L.J. (2000) Biochemical interactions integrating Itk with the T cell receptor-initiated signaling cascade, *J Biol Chem*, **275**, 2219-2230.

Diella, F., Cameron, S., Gemund, C., Linding, R., Via, A., Kuster, B., Sicheritz-Ponten, T., Blom, N. and Gibson, T.J. (2004) Phospho.ELM: a database of experimentally verified phosphorylation sites in eukaryotic proteins, *BMC Bioinformatics*, **5**, 79.

Karlsson, T., Songyang, Z., Landgren, E., Lavergne, C., Di Fiore, P.P., Anafi, M., Pawson, T., Cantley, L.C., Claesson-Welsh, L. and Welsh, M. (1995) Molecular interactions of the Src homology 2 domain protein Shb with phosphotyrosine residues, tyrosine kinase receptors and Src homology 3 domain proteins, *Oncogene*, **10**, 1475-1483.

Manke, I.A., Nguyen, A., Lim, D., Stewart, M.Q., Elia, A.E. and Yaffe, M.B. (2005) MAPKAP kinase-2 is a cell cycle checkpoint kinase that regulates the G2/M transition and S phase progression in response to UV irradiation, *Mol Cell*, **17**, 37-48.

Martins, L.M., Turk, B.E., Cowling, V., Borg, A., Jarrell, E.T., Cantley, L.C. and Downward, J. (2003) Binding specificity and regulation of the serine protease and PDZ domains of HtrA2/Omi, *J Biol Chem*, **278**, 49417-49427.

Nikolakaki, E., Du, C., Lai, J., Giannakouros, T., Cantley, L. and Rabinow, L. (2002) Phosphorylation by LAMMER protein kinases: determination of a consensus site, identification of in vitro substrates, and implications for substrate preferences, *Biochemistry*, **41**, 2055-2066.

Nishikawa, K., Sawasdikosol, S., Fruman, D.A., Lai, J., Songyang, Z., Burakoff, S.J., Yaffe, M.B. and Cantley, L.C. (2000) A peptide library approach identifies a specific inhibitor for the ZAP-70 protein tyrosine kinase, *Mol Cell*, **6**, 969-974.

Peri, S., Navarro, J.D., Kristiansen, T.Z., Amanchy, R., Surendranath, V., Muthusamy, B., Gandhi, T.K., Chandrika, K.N., Deshpande, N., Suresh, S., Rashmi, B.P., Shanker, K., Padma, N., Niranjan, V., Harsha, H.C., Talreja, N., Vrushabendra, B.M., Ramya, M.A., Yatish, A.J., Joy, M., Shivashankar, H.N., Kavitha, M.P., Menezes, M., Choudhury, D.R., Ghosh, N., Saravana, R., Chandran, S., Mohan, S., Jonnalagadda, C.K., Prasad, C.K., Kumar-Sinha, C., Deshpande, K.S. and Pandey, A. (2004) Human protein reference database as a discovery resource for proteomics, *Nucleic Acids Res*, **32**, D497-501.

Songyang, Z., Carraway, K.L., 3rd, Eck, M.J., Harrison, S.C., Feldman, R.A., Mohammadi, M., Schlessinger, J., Hubbard, S.R., Smith, D.P., Eng, C. and et al. (1995) Catalytic specificity of protein-tyrosine kinases is critical for selective signalling, *Nature*, **373**, 536-539.

Songyang, Z., Fanning, A.S., Fu, C., Xu, J., Marfatia, S.M., Chishti, A.H., Crompton, A., Chan, A.C., Anderson, J.M. and

Cantley, L.C. (1997) Recognition of unique carboxyl-terminal motifs by distinct PDZ domains, *Science*, **275**, 73-77.

Songyang, Z., Lu, K.P., Kwon, Y.T., Tsai, L.H., Filhol, O., Cochet, C., Brickey, D.A., Soderling, T.R., Bartleson, C., Graves, D.J., DeMaggio, A.J., Hoekstra, M.F., Blenis, J., Hunter, T. and Cantley, L.C. (1996) A structural basis for substrate specificities of protein Ser/Thr kinases: primary sequence preference of casein kinases I and II, NIMA, phosphorylase kinase, calmodulin-dependent kinase II, CDK5, and Erk1, *Mol Cell Biol*, **16**, 6486-6493.

Songyang, Z., Shoelson, S.E., Chaudhuri, M., Gish, G., Pawson, T., Haser, W.G., King, F., Roberts, T., Ratnofsky, S., Lechleider, R.J. and et al. (1993) SH2 domains recognize specific phosphopeptide sequences, *Cell*, **72**, 767-778.

Songyang, Z., Shoelson, S.E., McGlade, J., Olivier, P., Pawson, T., Bustelo, X.R., Barbacid, M., Sabe, H., Hanafusa, H., Yi, T. and et al. (1994) Specific motifs recognized by the SH2 domains of Csk, 3BP2, fps/fes, GRB-2, HCP, SHC, Syk, and Vav, *Mol Cell Biol*, **14**, 2777-2785.

Sparks, A.B., Rider, J.E., Hoffman, N.G., Fowlkes, D.M., Quillam, L.A. and Kay, B.K. (1996) Distinct ligand preferences of Src homology 3 domains from Src, Yes, Abl, Cortactin, p53bp2, PLCgamma, Crk, and Grb2, *Proc Natl Acad Sci U S A*, **93**, 1540-1544.

Wang, H.Y., Lin, W., Dyck, J.A., Yeakley, J.M., Songyang, Z., Cantley, L.C. and Fu, X.D. (1998) SRPK2: a differentially expressed SR protein-specific kinase involved in mediating the interaction and localization of pre-mRNA splicing factors in mammalian cells, *J Cell Biol*, **140**, 737-750.

Yaffe, M.B., Leparc, G.G., Lai, J., Obata, T., Volinia, S. and Cantley, L.C. (2001) A motif-based profile scanning approach for genome-wide prediction of signaling pathways, *Nat Biotechnol*, **19**, 348-353.
